# The integrative metabolomic-transcriptomic landscape of glioblastome multiforme

**DOI:** 10.18632/oncotarget.16544

**Published:** 2017-03-24

**Authors:** Dieter Henrik Heiland, Jakob Wörner, Jan Gerrit Haaker, Daniel Delev, Nils Pompe, Bianca Mercas, Pamela Franco, Annette Gäbelein, Sabrina Heynckes, Dietmar Pfeifer, Stefan Weber, Irina Mader, Oliver Schnell

**Affiliations:** ^1^ Department of Neurosurgery, Medical Center, University of Freiburg, Freiburg, Germany; ^2^ Institute of Physical Chemistry, Faculty of Chemistry and Pharmacy, University of Freiburg, Freiburg, Germany; ^3^ Department of Hematology, Oncology and Stem Cell Transplantation, Medical Center, University of Freiburg, Freiburg, Germany; ^4^ Department of Neuroradiology, Medical Center, University of Freiburg, Freiburg, Germany; ^5^ Faculty of Medicine, University of Freiburg, Freiburg, Germany

**Keywords:** metabolomics, transcriptomics, network analysis, glioblastoma multiforme, WGCNA

## Abstract

The purpose of this study was to map the landscape of metabolic-transcriptional alterations in glioblastoma multiforme. Omic-datasets were acquired by metabolic profiling (1D-NMR spectroscopy n=33 Patient) and transcriptomic profiling (n=48 Patients). Both datasets were analyzed by integrative network modeling. The computed model concluded in four different metabolic-transcriptomic signatures containing: oligodendrocytic differentiation, cell-cycle functions, immune response and hypoxia. These clusters were found being distinguished by individual metabolism and distinct transcriptional programs. The study highlighted the association between metabolism and hallmarks of oncogenic signaling such as cell-cycle alterations, immune escape mechanism and other cancer pathway alterations. In conclusion, this study showed the strong influence of metabolic alterations in the wide scope of oncogenic transcriptional alterations.

## INTRODUCTION

Glioblastoma multiforme (GBM) is the most common primary malignant brain tumor in adults, with an annual incidence of 3–4 cases per 100 000 people in Europe [1, 2] and the United states [3]. In spite of the best available treatment, the prognosis for patients with GBM is poor, with a median survival of not more than 14–16 months [4–8]. During the last decade, numbers of novelties in cancer development, genetic and metabolic alterations were discovered in the context of glioma [9]. Most notably, altered metabolism was described as a hallmark of cancer development and frequently occurs among different cancer types [10]. Furthermore, tumorigenesis is driven by the reprogramming of cellular metabolism, which derives directly or indirectly from genetic or epigenetic alterations [10]. The recently published revised WHO classification of brain tumors accounts for genetic alterations highlighting in gliomas especially the IDH mutation [11]. In glioma, the “Glioma-CpG island methylator phenotype“(G-CIMP) has been found to arise as a consequence of tumor-associated metabolic alterations [12, 13]. Usually, isocitric acid is transformed into α-ketoglutaric acid by IDH1/2. In case of a catalytic located *IDH1/2* mutation, isocitric acid is converted into the oncometabolite 2-hydroxyglutaric acid (2-HG) [12, 13]. Accumulation of 2-HG reshapes the tumor methylome and constitutes the G-CIMP [12, 13]. Interestingly, patients affected by the *IDH1/2* mutation reveal a better clinical course compared to non-mutated patients [14]. In particular, the IDH1/2 mutation and its metabolic changes pinpointed the strong coherence between metabolic and genetic alterations and highlighted the relevance of tumor-metabolism in glioblastoma multiforme.

### Metabolic/genetic profiling in glioma

The first observations on metabolic alterations in gliomas were made in ^1^H-magnetic resonance spectroscopy (H-MRS) some decades ago [15, 16]. Aided by array-based molecular methods and improved analytic approaches, new studies in cancer cell metabolism were expanded to the understanding of the mechanisms and functional consequences of tumor-associated metabolic alterations. Nuclear magnetic resonance (NMR) and ^1^H-MRS are established tools for metabolic profiling *in-vivo* or *ex-vivo*. A recent study analyzed nine glioma-cell lines by NMR spectroscopy [17]. The metabolic profiles were compared to publicity available gene expression data of these cell-lines. Four distinct profiles were detected, which were classified according to specific metabolic alterations [17]. In another study, the metabolic/proteomic signature of glioma-cell-lines was investigated. Eight metabolites were detected by NMR and compared to RPPA (reverse-phase protein array) proteomic analysis and expression data. The cell-lines were then clustered into “full-stem” and “restricted stem” subgroups, based on their stem-cell capacity [18]. Moreover, in a study performed by Chinnaiyan et al., 2012, where metabolic analysis was performed by liquid chromatography, altered anaerobic metabolism in the mesenchymal subgroup described by Verhaak and Phillips [19, 20] was shown. Pantel et al., 2014 analyzed tumor heterogeneity by single-cell RNA sequencing and investigated 4 subgroups of transcriptomic profiles, which co-existed within the same tumor [21]. The profiles were named by dominant biological function as oligodendrocytic differentiation, cell-cycle, immunoresponse and hypoxia [21]. Therefore, environmental conditions as hypoxia with its metabolic alteration may influence tumor heterogeneity and associated expression profiles in GBM and other cancers [22–24].

The purpose of this study was to integrate metabolomic and transcriptomic data by comprehensive network-based modeling. This analytic approach attempts to improve the knowledge about interacting regulation mechanism between metabolic and transcriptional alteration in glioblastoma multiforme.

## RESULTS

### Workflow and overview of combined methods

**Figure 1:**
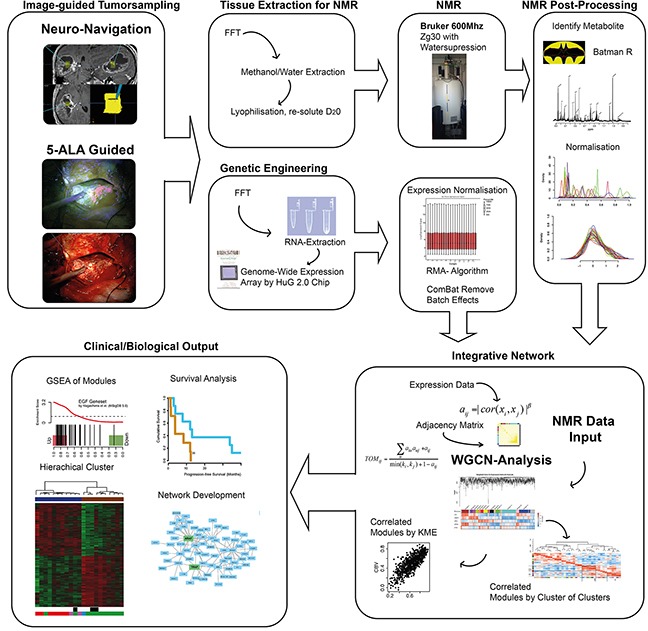
The figure reveals the workflow and data processing of the “in-house” pipeline This semi-automated analysis served as a robust method for integrative analysis of metabolic and genetic data.

This study contained a pipeline including several steps from, tissue sampling guided by neuronavigation, genetic and metabolic engineering up to bioinformatic analysis. In short, tissue samples were subjected to a combined genetic and spectroscopic analysis. Metabolites from 1H NRM spectroscopy and genetic expression data were normalized. Classical analysis (Consensus Cluster (k=5), unsupervised clustering and survival Analysis) of transcriptomic and metabolic data was performed separately on both datasets. For data integration, a network analysis using a topological overlapping measurement identified genetic modules [25]. Associations of each gene and the traits of interest (metabolites) were quantified by defining Gene Significance (GS) as the correlation between each gene and metabolite. Additionally, module membership (MM) for each gene was measured by correlation of module eigengene and the gene expression profile. The intramodule connectivity (kME) was calculated using the GS and MM measures. Therefore, genes were defined by its correlation with specific metabolites as well as its importance (ranked within its module membership). The kME allowed an identification of those genetic modules, which were associated to specific intracellular metabolism. All steps of the workflow were illustrated in Figure 1.

### Separate metabolic and transcriptomic profiling of glioblastoma multiforme

56 patients with neuronavigation-guided intraoperative sampling, available fresh frozen tissue samples and a histological confirmed glioblastoma multiforme were included in this study. RNA preparation and transcriptomic profiling was performed in 48 cases (low RNA quality in 8 cases), a total number of 38 tissue samples were analyzed by NMR (low tissue quality in 8 cases, low metabolite quality in 10 cases) and 4 samples not achieved post-processing quality criteria (fitting not successful).

**Figure 2:**
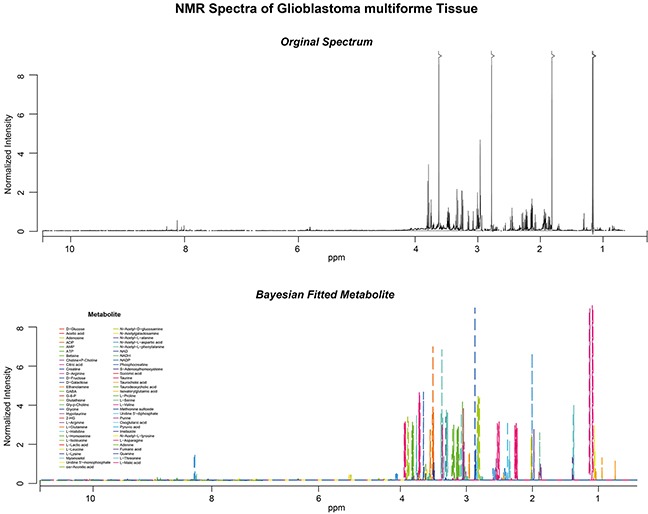
High resolutionNMR-Spectra of one patient including a raw spectra (upper panel) and fitted curves (lower panel)

**Figure 3:**
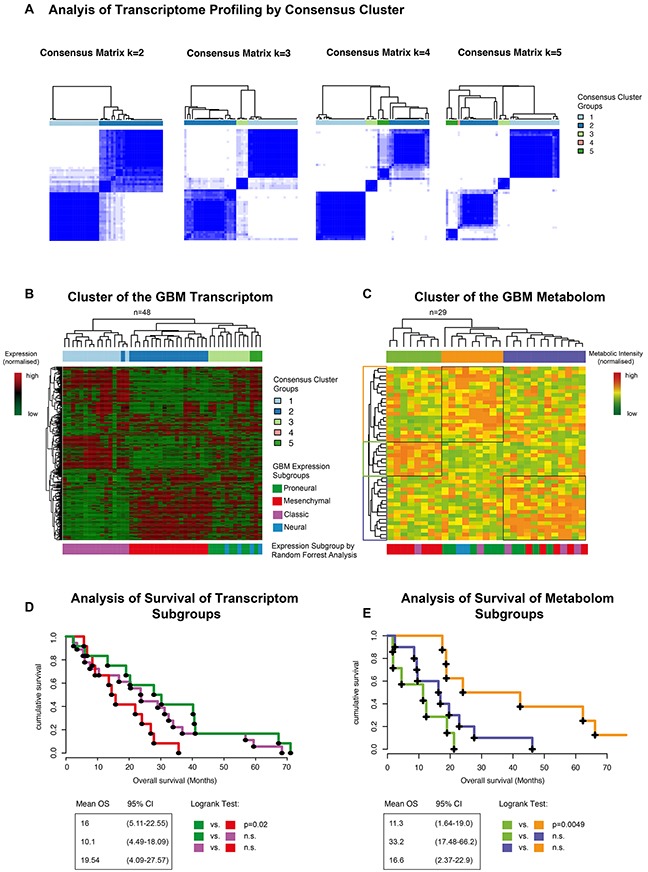
**(A)** Consensus cluster of expression data (n=48) revealed 4 cluster groups, which were summarized in an unsupervised clustering **(B)**. The bar below the heatmap indicated the expression subgroups (identified by random forest analysis). **(C)** Unsupervised hierarchical cluster of normalized metabolite values. Bars below the heatmap describe the expression subgroup of each patient. **(D-E)** Survival analysis of all clustergroups (derived from transcriptome **(D)** and metaboliom **(E)**) shows a significantly different overall survival with a more favorable outcome for the proneural subgroup of proneural-associated metabolic cluster.

Raw spectra (Figure 2, upper panel) were processed by a Bayesian-algorithm implemented in the R-software packed “Batman” (Figure 2, lower panel, detailed description in the method part). An unsupervised hierarchical clustering of the normalized metabolic intensity values of all tissue samples identified three clusters with distinct metabolic profiles (Figure 3C). Cluster yellow was associated to the proneural subgroup and revealed a significant better clinical course in comparison to the other cluster groups. Cluster green showed an association to the mesenchymal expression group and showed a poor clinical course. In comparison to the separate metabolic analysis the transcriptomic profiles of 48 patients were explored. A consensus clustering showed 3 main subgroups, which were confirmed by unsupervised hierarchical clustering (Figure 3A–3B). The expression subgroups of each patient were predicted by a random forest algorithm, which based on a training dataset (TCGA data set of 484 patients) with implemented subgroups by Verhaak (Figure 3B bottom panel). Three subgroups were found with distinct transcriptomic profiles. The proneural (PN) and neural (N) group was not separately grouped weather classical (CL) and mesenchymal (MES) samples grouped into distinct cluster. A survival statistics revealed no strong differences between all subgroups, only the PN subgroup revealed a significant improved survival compared to the MES subgroup. This analysis showed a clear separation of transcriptome and metabolic profiles into the described subgroups of Verhaak. To quantify these correlations an integrative analysis of both omic-datasets was performed.

### Combined integrative network-based analysis of metabolic and transcriptome profiling

**Figure 4:**
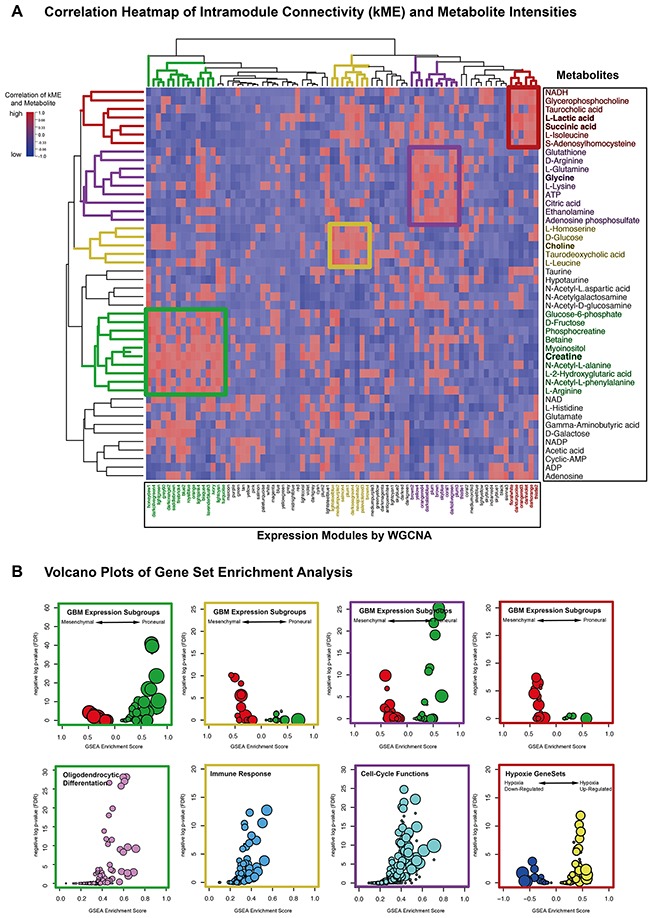
**(A)** Unsupervised hierarchical cluster of correlation coefficients (kME and normalized metabolite values). High correlations was colored in red, low correlation in blue. **(B)** Gene set enrichment analysis plots summarized enrichment scores of indicated biological functions. Enrichment Scores (ES) and p-values of all expressions modules (derived from WGCNA) were illustrated in a volcano plot. On the y-axis, the negative logarithm of GSEA p-values were presented, the x-axis contained ES-values. The size of each point indicated the level of gene set enrichment.

For further characterizing of each metabolite, a gene expression network with integrated metabolic data was build. Genes were summarized in 79 expression modules. Module-containing genes were ranked by their intramodule connectivity and correlated to each metabolite. These correlation coefficients were analyzed by an unsupervised hierarchical clustering (Figure 4A). Highly correlated metabolites and expression modules revealed four specific subgroups colored in green (Cluster I), yellow (Cluster II), violet (Cluster III) and red (Cluster IV).

### Cluster I (green): proneural/oligo-related cluster

Cluster I contained 10 metabolites and 16 expression modules. These expression modules were analyzed by gene set enrichment analysis (GSEA, detailed description in the method part). The results of the GSEA were summarized in a volcanoplot (Figure 3B). This plot illustrated each enrichment-score and the corresponded negative log of FDR p-values of each module within cluster 1. Most expression modules that were presented in cluster I significantly enriched proneural genes. In addition, oligodendrocytic genes were also highly enriched in most of the expression modules summarized in cluster I (Figure 4B). Creatine, was identified as “key-metabolite” defined as highest total kME correlation in each cluster group, which was significantly associated with all expression modules contained in cluster I.

### Cluster II (yellow): immune-related cluster

Cluster II comprised 5 metabolites and 8 expression modules. A GSEA was used for further functional characterization. An analysis of the known glioblastoma multiforme expression subgroups showed a stronger overall enrichment for the mesenchymal gene set. Interestingly, immune response and activation of the immune system were highly enriched in cluster (Figure 4B). The key-metabolites of this cluster were phosphocholine and choline being significantly correlated to all related expression modules.

### Cluster III (violet): cell-cycle-related cluster

Cluster III included 9 metabolites and 11 expression modules. Four expression modules showed a strong association with the proneural subgroup, while others enriched mesenchymal genes. Further analysis of other biological functions revealed a strong connection with cell-cycle functions including regulation of Phase M2, DNA repair mechanism and regulation of mitosis (Figure 4B). The metabolite glycine was identified as the key-metabolite of this cluster. It was significantly correlated to all related expression modules.

### Cluster IV (red): hypoxia/mesenchymal-related cluster

Cluster IV contained 7 metabolites and 6 expression modules. A strong enrichment of mesenchymal genes was found by GSEA. Only one expression module showed a non-significant enrichment of proneural genes. Key-metabolite of this cluster was lactate, which is a known agent of anaerobic metabolism (Figure 4B). Interestingly, expression modules of cluster IV highly enriched more genes that were up-regulated in hypoxic conditions than those being down-regulated.

**Figure 5:**
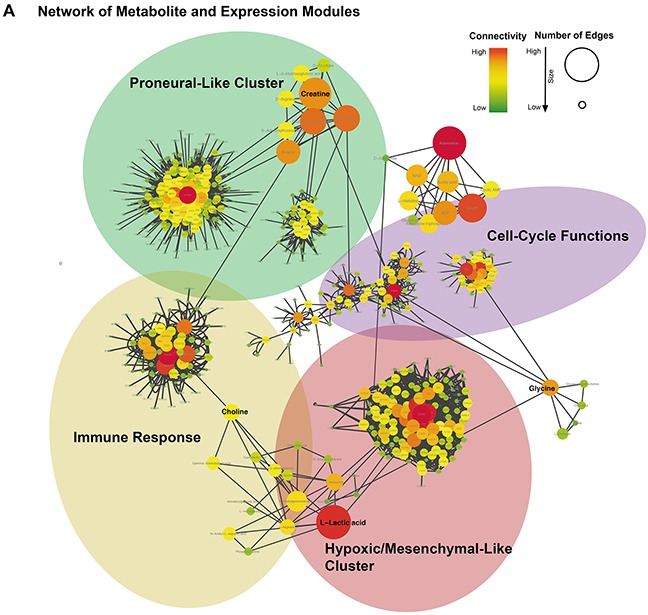
**(A)** Integrative network of metabolite and expression modules (WGCNA). Size and color indicated the importance of each gene/metabolite in the network. Hub-genes/metabolites were colored in red.

All connections between metabolite and correlated genes were given in a connectivity-weighted network and illustrated in Figure 5. This scheme represents the relationship between the metabolites and the genetic modules. It is apparent from this that some metabolites, such as myo-inositol and lactate, also have connections to further clusters than only to those identified primarily.

### Integrative interpretation of clusters

**Figure 6:**
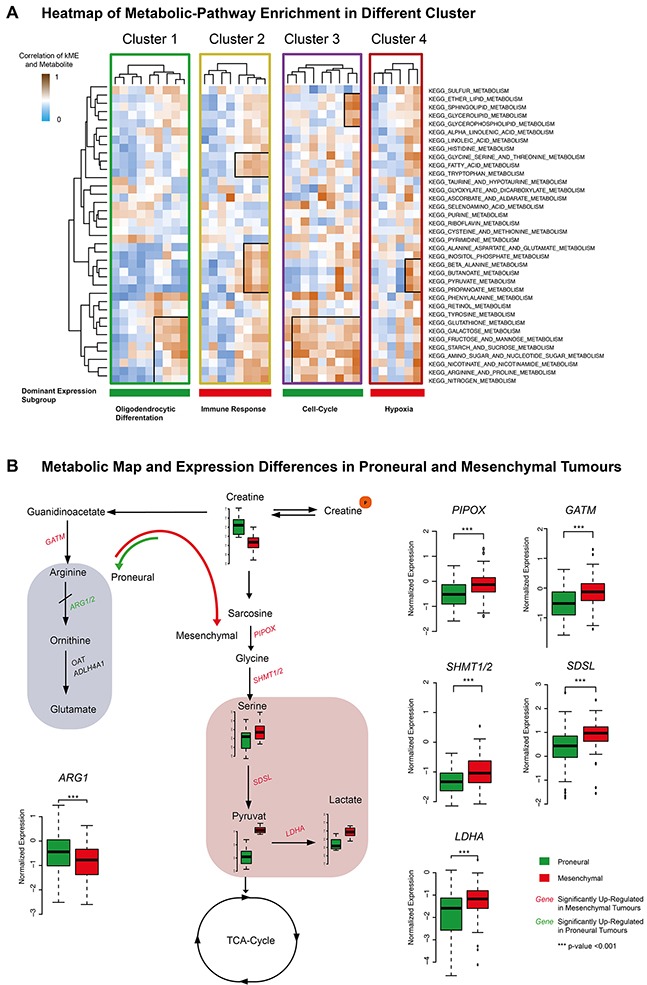
**(A)** KEEG metabolism-pathway of Cluster 1-4 was illustrated. The exclusive and overlapping enriched pathways were marked. The color code at the bottom showed the dominant expression subgroup and indicated the functional subgroup. **(B)** Map of metabolic differences on single-metabolite level and expression of enzymes belonging to the mapped metabolic pathways. *** p<0.001, ** p<0.01, *p<0.05.

Individual expression modules of each identified cluster (1-4) were separately analyzed by gene-set-variation analysis (GSVA) to find highly enriched metabolic-pathways (KEEG) (Figure 6A). Cluster 1 and 3 dominantly expressed proneural genes. These clusters showed an overlap of enriched metabolic pathways (Figure 6A), including arginine, proline-, glutathione-, and amino-sugar-metabolism. Cluster 3, which contained cell-cycle functions, exclusively enriched glycerolipid- and sphingolipid metabolism. Cluster 2 and 4 highly enriched mesenchymal genes and were characterized as immune response- or hypoxia-related. These clusters showed an overlap of metabolic pathways (pathways in line 22 – 25 on Figure 6A), spotlighted by the pyruvate metabolism. Most notably, Cluster 2 contained expression modules, which enriched dominantly genes of the immune response and exclusively enriched the tryptophan metabolism.

A map of metabolites associated with the proneural subtype (creatine) including the creatine-, arginine-, glycine-, serine- and pyruvate metabolism as illustrated in Figure 6B. Enzymes of the creatine degradation were significantly stronger expressed in mesenchymal samples, while enzymes of the arginine-proline metabolism were up-regulated in proneural tumors (Figure 6B).

## DISCUSSION

Gene expression and cell metabolism are frequently altered in glioblastoma and support cell proliferation, epigenetic alterations and tumor aggressiveness. In 2010, Verhaak proposed four glioblastoma multiforme subclasses by expression profiling [19]. These subgroups were characterized by specific gene sets, which were exclusively expressed in each subclass. Patel et al. (2014) showed a strong variance of the Verhaak expression subgroups based on single-cell sequencing of up to 100 cells within the same tumor [21]. Intratumoral heterogeneity within those subgroups could be demonstrated, and four transcriptional modules were additionally identified on single cell lines within the same tumor (Hypoxia, Immune, Oligo, Cell Cycle). These metabolic profiles reflect the final downstream product of gene transcription [26]. Therefore the purpose of this study was to combine genetic and metabolomic information to identify the transcriptomic-metabolomic landscape of glioblastoma multiforme, which mirrors the up- and downstream products of cellular regulation mechanisms.

In the first cluster analysis containing only spectral intensities, a significant association of metabolism to the Verhaak expression subgroups was found (Figure 3C). This connection was to be expected and has already been described in the literature [27]. In the next step, a network model was developed that integrates functional expression modules and correlated metabolites of GBM. This model resulted in four different cluster-groups, illustrated in Figure 4. Each cluster was interpreted in the context of its dominant underlying biological functions and contained: oligodendrocytic differentiation (Cluster 1), cell-cycle (Cluster 3), immune response (Cluster 2) and hypoxia (Cluster 4). Cluster 2 (immune response) and 4 (hypoxia) highly enriched mesenchymal genes. They showed an overlap of their enrichment of metabolic-pathway signatures, which highlight the pyruvate metabolism. Pyruvat plays an important role by connecting anaerobic and aerobic metabolism [28]. The immune response related cluster additionally enriched exclusively the tryptophane metabolism. A study by Moffett et al., 2003 described the major role of the tryptophane metabolism in several immune functions including the immune escape mechanism [29]. The key metabolite of the immune response-related cluster was choline. Although choline-containing compounds are generally discussed to be involved within membrane [30] turnover, other authors discussed the choline-containing compound to be related to immune response in crap [31].

The key metabolite of the hypoxia-related cluster was L-lactic acid. As generally known, lactate acid is used for energy metabolism in hypoxic environment [10, 27]. Hypoxic conditions were also known for a proneural-to-mesenchymal transition, which revealed strong coherence between metabolic environment and transcriptional regulation mechanism [32]. The metabolic and transcriptional differences of the pyruvate and lactate metabolism are presented in Figure 6. Our results point to the fact that metabolic alterations may drive the appearance of specific glioblastoma multiforme expression subclasses as a specific adaption upon microenvironmental conditions.

Clusters 1 and 3 dominantly enriched proneural genes and therefore belonged to the proneural subtype. Furthermore, cluster 1 showed a strong enrichment of oligodendrocytic differentiation. The key-metabolite was creatine, which is in line with the literature [33], where creatine was found to be highly associated with increased expression of proneural genes in a MRS *in-vivo* study [33]. Additionally, proneural samples were typically characterized by an up-regulation of oligodendrocytic genes as *OLIG1/2* [19]. The cluster 3 contained cell-cycle functions and showed an exclusive enrichment of the glycerolipid and sphingolipid pathway. This metabolic alteration is well known and highly associated with G0/G1- cell-cycle functions and other parts of cell-cycle regulation [34]. Its key-metbolite was glycine, which has several unspecific functions and could metabolized in several substrates (KEGG). A specific connection between glycine and cell cycle function has not been reported so far.

The study by Patel et al., 2014 analyzed single-cell transcriptomic profiles of glioblastoma multiforme within the same tumor. All profiles were summarized in four clusters containing: oligodendrocytic differentiation, cell-cycle, immune response and hypoxia. These results were totally in-line with our reported findings. In fact, tumor metabolism is highly inhomogeneous within the same tumor and our study was limited by only single biopsies, taken from the contrast-enriched part. In contrast to limited analysis by metabolomic/transcriptomic data alone, an integrative approach of metabolomic-transcriptomic features allowed to capture all subgroups being described by Patel et al. This is attributed to the fact, that integrative approach mirrors the intracellular functional processes better than transcriptomic profiling alone.

All these findings confirm a strong link between metabolic alterations and specific expression subclasses. Known metabolic alterations (*IDH1/2* mutation) are able to reshape the tumor methylome and may also affect specific traits of tumor gene expression. For the first time, our study described metabolic alterations, which were related to specific expression subtypes of glioblastoma multiforme. Therefore, our results point to the fact that metabolic alterations may drive the appearance of specific glioblastoma multiforme expression subclasses as a specific adaption upon microenvironmental conditions.

Finally, these findings highlight the strong mutual dependence of transcriptional oncogenic alterations and glioblastoma metabolism. The global network, which described the landscape of transcriptional/metabolomics alterations (Figure 5) revealed lots of new findings beyond the reported metabolic connections. These GBM specific alterations could be used to explore further new therapeutic strategies.

### Limitations of the study

This study has some limitations. First, the small sample size and the disregarded heterogeneity could have lead to false-positive associations or confounder effects. Second, samples were guided by intraoperative neuronavigation without correction for brain shift. H&E stainings confirmed the occurrence of tumor in each biopsy. Additionally, tissue samples also contained non-tumor cells (1-10%), which could have an impact on global metabolomics/transcriptomic profiling. Conservative statistical methods with corrections for multiple testing at each level of analysis were applied. Only corrected p-values (Bonferroni, Benjamini-Hochberg) were reported for the sake of robustness. Nevertheless, these findings have to be confirmed in a larger cohort of patients.

### Conclusion

This study displayed the landscape of metabolic-transcriptomic alterations in glioblastoma multiforme. Our results point to the fact that metabolic alterations may drive the appearance of specific glioblastoma multiforme expression subclasses. Additionally, we highlighted the association between metabolism and hallmarks of tumorigenesis such as cell-cycle dysfunctions or immune escape mechanisms. In conclusion, the strong influence of metabolic alterations in the wide scope of oncogenic transcriptional alterations.

## MATERIALS AND METHODS

### Workflow and study design

An illustration of the following workflow is shown in Figure 1. A detailed description of each analytic step is presented below.

### Patients

For this prospective study we included 48 patients with primary glioblastoma multiforme WHO grade IV (without known lower-grade leasion in the patients history), who underwent surgery at the Department of Neurosurgery of the Medical Center, University of Freiburg between 2012 and 2016. The local ethics committee of the University of Freiburg approved data evaluation, imaging procedures and experimental design (protocol 100020/09 and 5565/15). The methods were carried out in accordance with the approved guidelines. Written informed consent was obtained from all patients.

### Tissue collection and histology

Tumor tissue was sampled from contrast enhancing regions identified by intraoperative neuronavigation (Cranial Map Neuronavigation Cart 2, Stryker, Freiburg, Germany) during tumor resection. The tissue was snap-frozen in liquid nitrogen immediately after resection and processed for further genetic/metabolic analysis. Tissue samples were fixed using 4% phosphate buffered formaldehyde and paraffin-embedded with standard procedures. H&E staining was performed on 4 μm paraffin sections using standard protocols. Immunohistochemistry was applied using an autostainer (Dako) after heat-induced epitope retrieval in citrate buffer. IDH1 mutation was assessed by immunohistochemistry using an anti-IDH1-R123H antibody (1:20, Dianova).

### 1H-NMR spectroscopy

Frozen tissue samples (n=48) were extracted by methanol-water (M/W) extraction. The M/W extraction was performed as described in Beckonert et al [35]. Extracts were disintegrated by sonication. Half of the extract was used to extract DNA and determine its total concentration (for normalization step-1, as described below). Only 38 samples achieved the quality criteria. The hydrophilic-phase was separated, lyophilized and resolved in deuteriumoxide (D_2_O). 600μl suspension was transferred to NMR-tubes for further NMR procedures. ^1^H-NMR was performed at the Institute of Physical Chemistry of the University of Freiburg. 1D-NMR spectra were performed on a Bruker Avance III HDX 600-MHz FT-NMR spectrometer (Rheinstetten, Germany), equipped with the probe: PABBO BB/19F-1H/D Z-GRD. Each single spectrum within the spectra was recorded with 2 dummy scans and 32 scans with 64k points in the time domain. The sweep width was set to 16.02 ppm with an offset of 4.691 ppm. This resulted in an acquisition time of 3.4 seconds for each scan with a dwell time of 52 microseconds. The relaxation delay was set to 2 seconds, so that the total acquisition time of each spectrum was 3 minutes and 5 seconds. Water-suppressed 1H NMR spectra were acquired by using a *zgesgp* sequence [36]. The FID was Fourier transformed and automatically phase corrected without any further zero filling or apodization. The spectra were manually calibrated by setting the peak of L-lactate acid at 1.310 ppm. All acquisition and processing of the spectra was performed with TopSpin 3.2 patchlevel 6. A total number of 29 spectra achieved high quality, which is necessary for complex post-processing.

### Post-processing and evaluation

Raw data was analyzed by “batman”, a R-software based tool for metabolite detection in complex spectra. The batman software fits a predefined list of metabolites by a Bayesian approach. A detailed description of the batman algorithm was given by Hao et al [37]. Four spectra did not archive quality criteria and were excluded from further analysis. Metabolite (spectral) intensities were used as further input for the integrative analysis.

### Data normalization

Data was normalized by a two-step normalization-approach. First, raw intensity values were normalized by the given DNA concentration of each patient to balance different tumor mass of each extract. Second, normalization was performed by a median-based normalization-algorithm.

### Genome-wide expression analysis

RNA of 48 patients was prepared using the RNAeasy kit (Qiagen). An amount of 1.5 μg RNA was obtained for expression arrays analysis. 8 samples did not achieve RIN>8.5 and could not used for further transcriptomic profiling. Arrays were performed by human gene ST 2.0 chip (Affymetrix). Raw data were processed, normalized and controlled by R software and the “affy” R-package. Different expression analysis and statistical testing (pairwise t-test) were performed by limma R-package.

### Identification of expression-subgroups

A consensus cluster analysis was performed by the implemented R package “ConsensusClusterPlus”, with a *kmax* of 10. The samples were dominantly split into 3 Clusters, which was also revealed by an unsupervised hierarchical clustering (Figure 3A–3B). Additionally, a random forest algorithm was used to identify the Verhaak expression subgroups for each sample (R-package: “randomForest”) [19]. Expression data of 484 TCGA samples were processed at level-3 and analyzed in the forest model as training data. The model showed a specificity of 94.3% and a sensitivity of 89.3%. Expression subgroups were predicted in the expression data of 48 patients based on the trained algorithm [38].

### Weighted gene co-expression network analysis and gene set enrichment analysis

The WGCNA analysis is a robust tool for integrative network analysis and was used in recent studies [39–41]. It is based on a scaled-topology-free based network approach and uses the topological overlapping measurement to identify corresponding modules as shown in Figure 1. These modules were analyzed by their eigengene correlation to each metabolite. The correlation of the intramodule connectivity (kME) and metabolites was used as input for a “Cluster of Clusters analysis”. This analysis integrates expression modules and metabolites, which present equal correlation values (kME and metabolite intensity values). A detailed description of WGCNA is given in Heiland et al. 2016 [33].

### Gene set enrichment analysis (GSEA) and gene set variation analysis (GSVA)

A permutation-based pre-ranked Gene Set Enrichment Analysis (GSEA) was applied to each module to verify its biological functions and pathways [42]. The predefined gene sets of the Molecular Signature Database v5.1 were taken. Enrichment score was calculated by the rank order according to normalized intramodule connectivity of each gene in the expression module [42]. For significant enrichment, p-values were adjusted by FDR. Gene Set Variation Analysis (GSVA) was performed by the GSVA package implemented in R-software. The analysis based on a non-parametric unsupervised approach, which transformed a classic gene matrix (gene-by-sample) into a gene set by sample matrix resulted in an enrichment-score for each sample and pathway [43].

### Statistic analysis

Normalized intensity values were clustered in an unsupervised hierarchical clustering. Cluster analysis was performed in r-software and affiliated packages. The Kaplan–Meier method was used to provide median point estimates and time-specific rates. The Hazard-Ratio (HR) was calculated by Cox-Regressions tests.
